# Pluronic^®^ F127 Polymeric Micelles as Nanocarriers for Pentamidine: Improving Safety and Biological Efficacy Against *Leishmania major*

**DOI:** 10.3390/ijms27031300

**Published:** 2026-01-28

**Authors:** Kristell Panta Quezada, Gustavo González-Gaitano, Paul Nguewa

**Affiliations:** 1 Department of Microbiology and Parasitology, Navarra Institute for Health Research (IdisNA), University of Navarra, 31080 Pamplona, Spain; kpantaqueza@alumni.unav.es; 2 Department of Chemistry, School of Science, University of Navarra, 31080 Pamplona, Spain

**Keywords:** leishmaniasis, micellar encapsulation, gene expression, pentamidine, Pluronic^®^ F127

## Abstract

Cutaneous leishmaniasis (CL) is a neglected tropical disease for which current chemotherapeutic options are limited by systemic toxicity (such as hepato-nephrotoxicity, arrhythmia, nausea, vomiting) and difficult administration regimens. Pentamidine (PTM), although effective, exhibits severe dose-limiting adverse effects. Polymeric micelles based on Pluronic^®^ F127 (F127) offer an attractive strategy to improve PTM delivery by enhancing solubility, reducing cytotoxicity, and enabling controlled release. Here, we developed PTM-loaded F127 polymeric micelles and performed a multidisciplinary evaluation combining physicochemical characterization, in vitro biological assays, and gene expression profiling. Dynamic light scattering, UV–visible absorption, fluorescence spectroscopy, and NMR confirmed micelle formation, PTM–polymer interactions, and temperature-dependent assembly. PTM-loaded micelles exhibited biorelevant nanoscale dimensions and preserved stability under physiological conditions. Biological assays demonstrated that F127 micelles markedly reduced PTM cytotoxicity in RAW264.7 macrophages while maintaining potent antileishmanial activity against *Leishmania major* promastigotes. RT-qPCR analysis revealed modulation of key pathways involved in redox homeostasis, oxidative stress, calcium regulation, apoptosis-like responses, and drug resistance, suggesting that micellar encapsulation influences both PTM bioavailability and parasite stress responses. Overall, PTM-loaded F127 micelles significantly improved the therapeutic index of PTM in vitro. These findings support the potential of F127 polymeric micelles as a promising nanocarrier platform for safer and more effective CL therapy.

## 1. Introduction

Leishmaniasis comprises a group of vector-borne diseases caused by protozoan parasites of the genus *Leishmania*, transmitted through the bite of infected female phlebotomine sand flies [1]. More than 12 million people are infected worldwide. In fact, this neglected tropical disease (NTD) is endemic in 99 countries mainly located in the Indian subcontinent, East Africa, and Brazil (visceral leishmaniasis) and in Latin America, Central Asia, the Middle East, and the Mediterranean Basin (cutaneous leishmaniasis). In addition, leishmaniasis also affects animals [2]. As mentioned above, the clinical spectrum includes cutaneous (CL), mucocutaneous (MCL), and visceral leishmaniasis (VL), with CL representing the most prevalent form and causing substantial morbidity. Despite an estimated 600,000–1,000,000 new CL cases annually, underreporting remains high, with fewer than 200,000 cases officially documented by the World Health Organization each year [3]. The primary hindrance in controlling leishmaniasis remains the lack of a clinically approved vaccine and the limitations of current pharmacological agents [4].

Current therapeutic strategies for leishmaniasis primarily involve systemic treatments with pentavalent antimonials, pentamidine (PTM), and amphotericin B [5]. However, these traditional treatments require prolonged parenteral administration and are associated with significant systemic toxicity (including hepatotoxicity, nephrotoxicity, arrythmias, prolonged QT interval, nausea, vomiting, rash, and local pain or abscess formation at the injection site), resulting in poor patient adherence and treatment interruptions.

The immunopathology of CL reflects a complex interplay between the parasite, the host’s immune system, bacteria from the human skin microbiota (or those causing superinfection of the ulcer), and the gut microbiota of the vector [6]. Early infection engages macrophages, dendritic cells, and neutrophils [7,8], initiating Toll-like receptor-9 (TLR-9)-dependent production of interleukin-12 (IL-12) and activation of natural killer (NK) cells. NK-cell-derived interferon-gamma (IFN-γ) and tumor necrosis factor-alpha (TNF-α) subsequently stimulate macrophages to generate reactive oxygen and nitrogen species that control intracellular parasite replication [8,9]. CD4^+^ Th1 cells amplify this response [10,11]. Conversely, *Leishmania* parasites deploy sophisticated mechanisms to counteract oxidative damage, relying on a unique trypanothione-based redox system absent in mammalian cells [12,13,14]. Key enzymes include gamma-glutamylcysteine synthetase (*γ-GCS*), trypanothione reductase (*TR*), glutathione peroxidase (*TDPX*), and tryparedoxin peroxidase (*TryP*) [15,16]. These proteins are critical for parasite survival and have emerged as promising drug targets. The inhibition of those enzymes or the downregulation of the corresponding encoding genes may significantly impair the parasite’s ability to withstand oxidative damage, dramatically reduce its infectivity, and can increase its sensitivity to drug treatments [12,13,16].

Additional pathways contribute to parasite fitness, including calcium homeostasis regulated by P-type ATPases (ATPase, *SERCA*) [17,18], membrane permeability mediated by Aquaglyceroporin 1 (*AQP1*) [19], mitochondrial respiration via cytochrome c oxidase subunits [20], and apoptosis-like cell death governed by metacaspases [21] and calpain-like proteases [22]. Finally, drug resistance remains a major challenge, largely mediated by the ATP-binding cassette (ABC), such a Multidrug-resistant protein A (*MRPA*) and Pentamidine Resistance Protein 1 (*PRP1*), which actively export drugs like antimonials and pentamidine, respectively [23,24].

**Figure 1:**
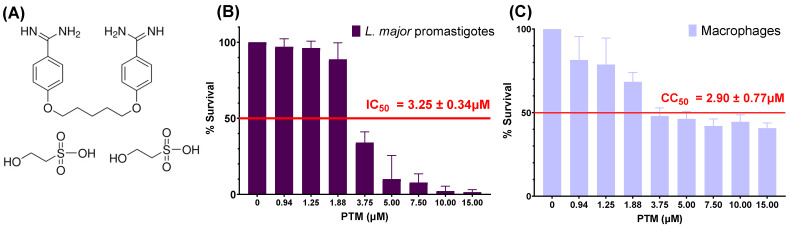
Pentamidine structure (**A**) and Biological activity (**B**,**C**). (**B**) Leishmanicidal activity of PTM in *L. major* promastigotes (*n* = 5). (**C**) Cytotoxicity of PTM on macrophages (*n* = 4). IC_50_ and CC_50_ values are provided as Mean ± SD.

PTM (Figure 1A), an aromatic diamidine, binds to proteins and nucleic acids including DNA [25], disrupts polyamine biosynthesis, inhibits RNA polymerase activity, and interferes with protein, nucleic acids, phospholipids, and folate synthesis [26]. Despite its potent leishmanicidal activity and potential immunomodulatory effects [27], PTM is associated with severe toxicities, including hypoglycemia (due to pancreatic islet cell damage), pancreatitis, hepatotoxicity, arrhythmias, prolonged QT interval, fatigue, dysgeusia, anorexia, nausea, vomiting, rash, nephrotoxicity, electrolyte imbalances, leukopenia, thrombocytopenia, and local pain or abscess formation at the injection site [26]. These limitations highlight the urgent need for alternative delivery strategies that preserve efficacy while reducing systemic exposure.

**Figure 2:**
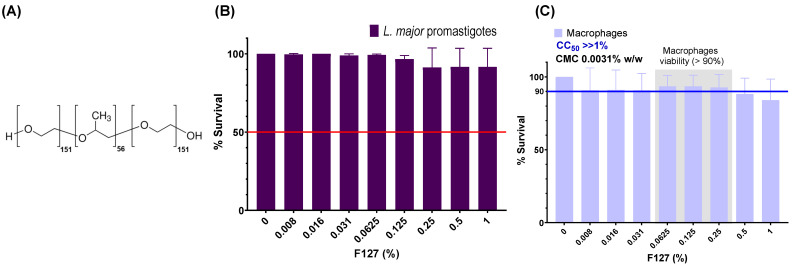
Pluronic^®^ (F127) structure (**A**) and its biological evaluation (**B**,**C**). (**B**) Analysis of its antileishmanial effect in *L. major* promastigotes and (**C**) Cytotoxicity of this polymeric excipient to macrophages. Data indicate mean ± SD from four independent experiments.

Polymeric micelles (PMs) formed by amphiphilic block copolymers have attracted considerable interest for drug delivery. Their core–shell architecture enables encapsulation of hydrophobic or amphiphilic drugs, improves solubility, prolongs circulation time, enhances stability, and reduces toxicity [28,29,30,31,32]. Pluronic^®^ F127 (F127) (PEO100-PPO69-PEO100) is a biocompatible triblock copolymer (Figure 2A) with a low critical micelle concentration (CMC 0.0031%), high biocompatibility, and proven safety [33]. F127 micelles may also undergo thermoreversible gelation at higher concentrations, offering opportunities for local depot-based delivery in dermal applications [34].

Given the challenges associated with PTM therapy, we hypothesized that encapsulating PTM within F127 micelles would enhance its physicochemical properties, mitigate its cytotoxicity, and improve its biological performance against *Leishmania major*. To test this hypothesis, we prepared PTM-loaded F127 micelles, evaluated their physicochemical properties, assessed antileishmanial activity and cytotoxicity, and investigated alterations in parasite gene expression associated with key metabolic and stress pathways.

## 2. Results

### 2.1. Biological Evaluation of Pentamidine and F127

Initial biological evaluations were conducted to characterize the individual effects of pentamidine and F127. PTM showed potent antileishmanial activity, alongside significant cytotoxicity towards macrophages (Figure 1). Figure 1B,C presents the dose–response curve for PTM after 48 h of exposure. Figure 1B illustrates the effect of the drug on *L. major* promastigotes, with a concentration-dependent decrease in parasite survival and an IC_50_ = 3.25 ± 0.34 μM. However, PTM also displays toxicity to macrophages (CC_50_ = 2.90 ± 0.77 μM) after 48 h of treatment (Figure 1C). These results underscore the narrow selectivity index (SI < 1) of PTM, as its effective antileishmanial concentration (3.25 μM) is quite close to its cytotoxic concentration (2.90 μM) in host macrophages, thereby highlighting the need for strategies to improve its safety profile.

Subsequently, the activity and cytotoxicity of F127 were also evaluated. F127 alone exhibited no significant leishmanicidal activity against *L. major* promastigotes (Figure 2B). Furthermore, its cytotoxicity was assessed against RAW264.7 macrophages over a 48 h period (Figure 2C). F127 demonstrated significantly lower cytotoxicity, with macrophage viability remaining above 90% even at F127 concentrations over 0.5%. The CC_50_ for F127 was found to be greater than 1%, confirming its high biocompatibility and low toxicity to macrophages within the tested range (Figure 2C). The reported CMC of F127 is 0.0031% *w*/*w* [33], indicating micelle formation at very low concentrations, well below its cytotoxic threshold. These results support the selection of F127 as a suitable polymer for drug delivery systems due to its favorable safety profile.

### 2.2. Physicochemical Characterization of F127 and Its Interaction with PTM

**Figure 3:**
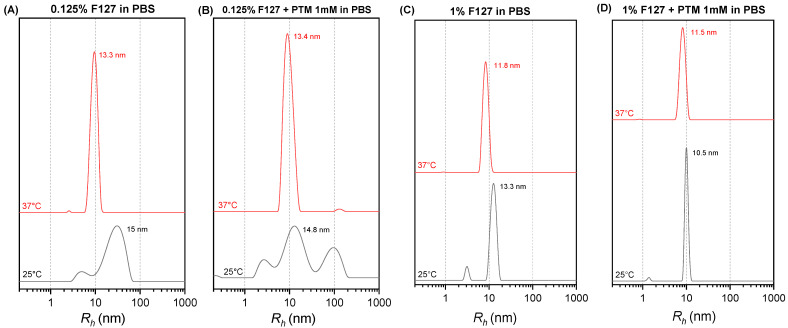
DLS intensity size distributions expressed in hydrodynamic radius obtained in PBS. (**A**) 0.125% F127 at 25 °C and 37 °C. (**B**) F127 and 1 mM PTM at 25 °C and 37 °C. (**C**) 1% F127 in PBS at 25 °C and 37 °C. (**D**) 1% F127 and 1 mM PTM in PBS at 25 °C and 37 °C.

DLS measurements were performed to evaluate the influence of the presence of PTM on the self-assembly of F127. Figure 3 shows the hydrodynamic radius (*R_h_*) distributions of 0.125% F127 micelles in PBS, both alone and in the presence of 1 mM PTM, at 25 °C and 37 °C. At 37 °C, both 0.125% F127 alone (Figure 3A) and with PTM (Figure 3B) exhibited sharp, monomodal peaks, indicating the formation of fairly monodisperse micelles. Specifically, for 0.125% F127 at 37 °C in PBS, the Rh is 13.3 nm. The micelle size remains largely unchanged (13.4 nm) upon the addition of PTM, although a noticeable increase in the width of the size distribution is observed, with polydispersity index (PDI) passing from 0.15 to 0.23.

The effect of increasing the polymer to PTM ratio has been studied. The size distribution for 1% F127 in PBS without PTM is shown in Figure 3C. At 37 °C (red line), a sharp, monomodal peak is observed at 11.8 nm, indicative of fully formed micelles. At 25 °C (black line), the distribution is broader, with a dominant peak at 13.3 nm. Figure 3D presents the DLS intensity size distributions for 1% F127 coencapsulating 1 mM PTM in PBS. Similarly to the surfactant alone, at 37 °C (red line), a sharp, monomodal peak centered at 11.5 nm is observed, consistent with stable micelle formation. At 25 °C (black line), a sharp, monomodal peak appears at 10.5 nm.

**Figure 4:**
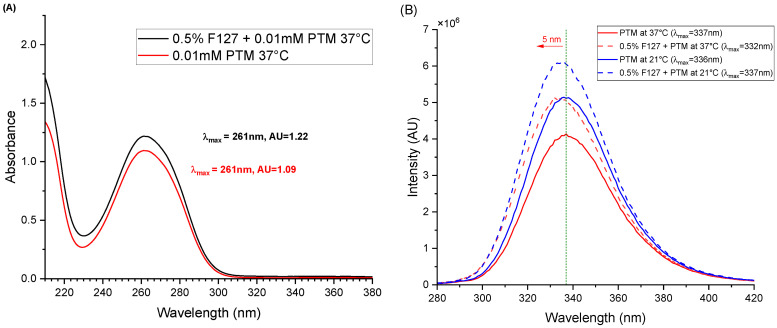
(**A**) UV–Visible absorption spectra of PTM in the absence and presence of 0.5% F127 at 37 °C. (**B**) Fluorescence spectra of PTM and F127 (0.5%) in PBS buffer, showing the blue shift and increase in intensity (AU) of the emission band.

The interactions between drugs and their carrier usually involve changes in the electronic environment that can be detected by electronic spectroscopy. Figure 4A presents the absorption spectra of PTM at 37 °C in the absence and presence of 1% F127. Both exhibit the characteristic absorption maximum of the drug at approximately 261 nm. The combination F127 and PTM results in an increase in the maximum of absorbance of PTM of approximately 11%, while no significant shift in the λ_max_ of the drug is detected. The effect of the copolymer is more noticeable when considering the fluorescence of the drug. The fluorescence emission spectra of PTM at the two temperatures considered, both in the absence and presence of 0.5% F127, are shown in Figure 4B. A notable increase in the intensity is observed in the combination of the drug at both 37 °C and 21 °C. Accompanying this intensity enhancement, a significant blue shift of 5 nm (λ_max_ shifts from 337 nm to 332 nm) is evident at 37 °C (Figure 4B). At 21 °C, the presence of F127 resulted in an emission wavelength that is virtually the same (337 nm), albeit with a slight emission increase (18%).

**Figure 5:**
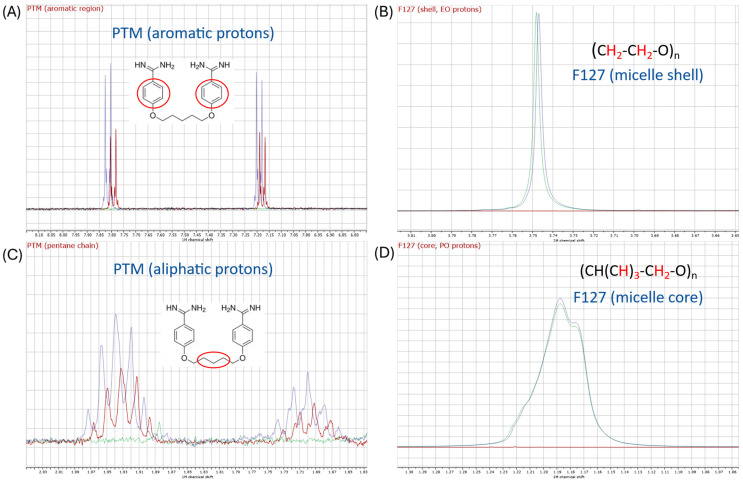
Selected regions of the ^1^H NMR spectra of PTM + F127 system at 37 °C in D_2_O. (**A**) 1 mM PTM aromatic region. (**B**) 2% F127 shell, EO protons. (**C**) 1 mM PTM pentane chain (**D**) 2% F127 core, PO protons. PTM, blue trace; F127 green trace; PTM + F127 red trace.

The interaction of PTM and F127 was also investigated using ^1^H NMR spectroscopy. Figure 5A shows the aromatic region of the proton spectrum of PTM at 37 °C in the absence (blue trace) and presence of 2% F127 (red trace), in which a small but representative downfield shift of the aromatic quadruplet (0.022 ppm) is observed compared to PTM alone. This shift also occurs in the signals of the aliphatic protons of the central part of the molecule (0.035 ppm, Figure 5C) but not in the isethionate counterion. Interestingly, the resonance at 1.19 ppm (green trace) corresponding to the hydrophobic PO residues, located at the core of the micelle, does not change in the presence of the drug, in contrast to the EO protons (3.75 ppm), which shift upfield 0.001 ppm. These changes indicate a modification of the magnetic environment of the drug when micelles are present, compatible with its incorporation in the aggregates.

**Figure 6:**
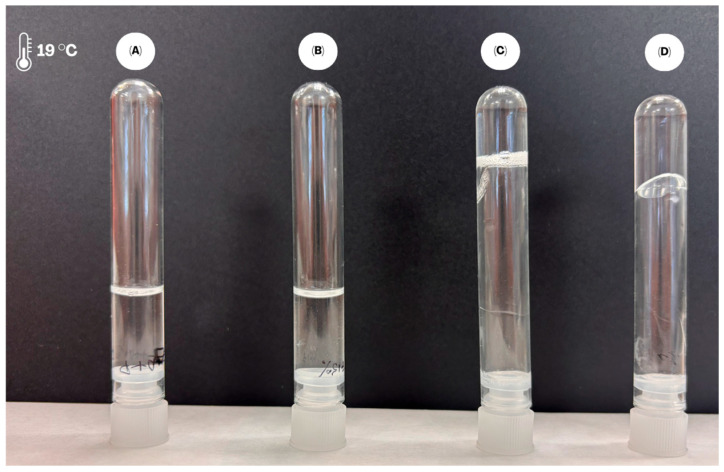
Impact of drug loading on the gelation of 15% and 20% F127. (**A**) 15% F127. (**B**) 15% F127 + PTM. (**C**) 20% F127. (**D**) 20% F127 + PTM.

Finally, based on the results of the reduced cytotoxicity of PTM and the preserved leishmanicidal activity obtained with the micellar system, the gelation behavior has been tested, as this characteristic is critical for the system’s in vivo performance (Figure 6). The dependence of gel formation on concentration and temperature was evaluated for F127 formulations in the presence of PTM. At 15% F127, the system remained as a viscous liquid throughout the entire temperature range from 10 °C to 61 °C, even in the presence of the drug. In contrast, at 20% F127, gel formation was observed starting at 19 °C, and this state was maintained up to 61 °C. The addition of PTM did not significantly alter the gelation transition temperature of the 20% F127 system, although a viscous liquid state was observed at 16 °C in the drug-loaded sample, suggesting a slight influence on viscosity at temperatures near the phase transition. This gelling behavior is consistent with the data described in the literature [35].

### 2.3. Biological Evaluation of Drug-Loaded Micelles

**Figure 7:**
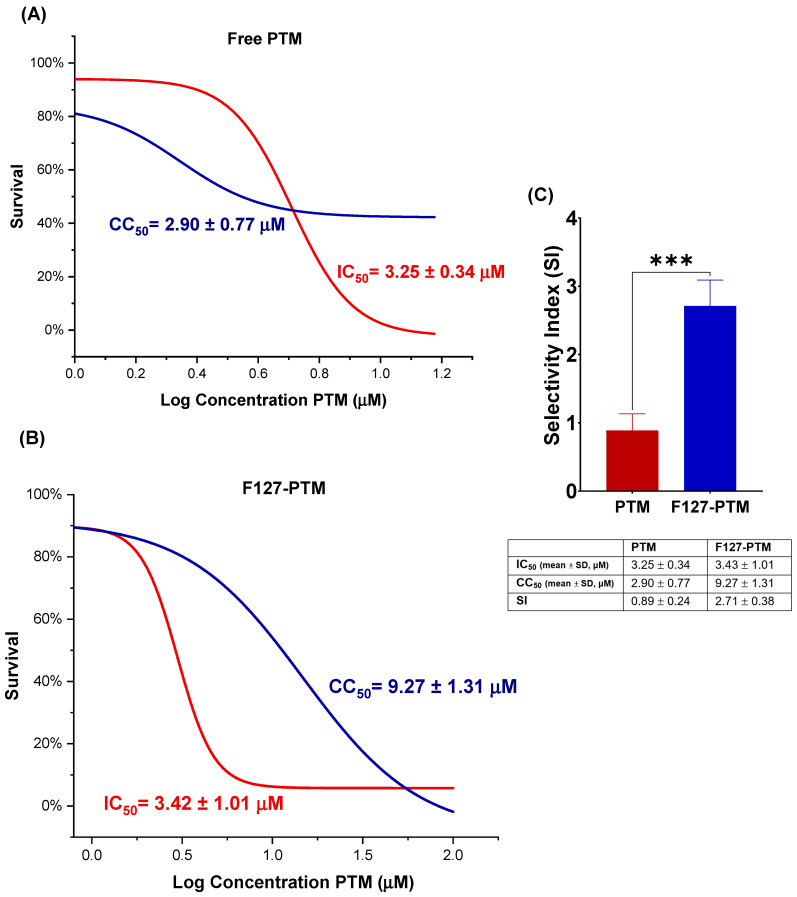
Antileishmanial activity and cytotoxicity of free and micellar PTM. (**A**) Dose–response curves of free PTM. (**B**) Dose–response curves of F127 (0.125%) + PTM (µM)—48 h (micellar formulation). Percentage of cell survival is plotted versus the logarithm of PTM concentration. Values are expressed as mean ± SD from four independent experiments. Solid lines represent the sigmoidal non-linear regression fit used to determine IC_50_ and CC_50_ values. (**C**) Selectivity Index (SI of PTM and F127-PTM) values are also provided as mean ± SD (*n* = 4). Significant differences are indicated (asterisks represent *p*-values: *** *p* < 0.001).

To assess the impact of micellar encapsulation on the antileishmanial activity and cytotoxicity of PTM, mixed micelle formulations were evaluated. The cytotoxicity of PMs composed of F127 (at 0.0625%, 0.125%, and 0.25%) with different concentrations of PTM (0.5 to 7 μM) was determined. Notably, formulations containing F127 at 0.125% combined with PTM showed a significantly increased CC_50_ (9.27 ± 1.31 μM) compared to PTM alone (2.90 ± 0.77 μM), indicating a substantial reduction of its cytotoxicity (Figure 7). This result suggests the successful attenuation of PTM cytotoxicity through micellar encapsulation within F127 systems. Figure 7A,B show the dose–response curves of PTM and its micellar formulation, F127-PTM. In antileishmanial assays, both formulations exhibited a concentration-dependent inhibitory effect. The micellar formulation retained potent antileishmanial activity, with IC_50_ = 3.42 ± 1.01 μM, comparable to that of the free PTM (3.25 ± 0.34 μM), demonstrating that micellar encapsulation does not compromise the antiparasitic efficacy of the drug.

In contrast, a marked improvement in the toxicity profile was observed in host cells (Figure 7B). Free PTM induced cytotoxicity at low concentrations, whereas F127-PTM produced a clear rightward shift in the cell viability curve, consistent with reduced host cell toxicity. Accordingly, the selectivity index (SI), calculated as the CC_50_/IC_50_ ratio, increased from 0.89 ± 0.24 for PTM to 2.71 ± 0.38 for F127-PTM, reflecting a significant enhancement in selectivity toward the parasite (*p* = 0.0002).

### 2.4. Gene Expression Profiling Analysis

**Figure 8:**
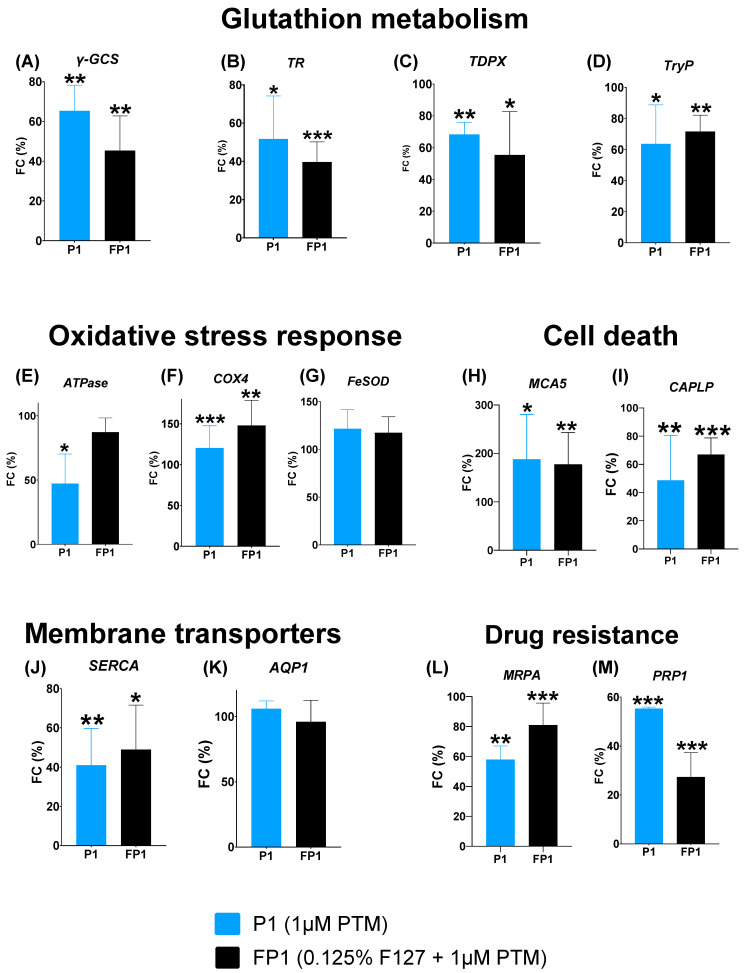
Analysis of gene expression profiling in *L. major* after treatment with free PTM (1 µM) and F127 micellar formulation (containing 1 µM PTM). Quantitative PCR was performed to assess expression levels of genes related to five key cellular pathways: glutathione metabolism (**A**–**D**), oxidative stress response (**E**–**G**), membrane transport (**H**,**I**), cell death (**J**,**K**), and drug resistance (**L**,**M**). Gene expression is represented as fold-change percentage, FC (%), relative to gene expression levels in untreated control (set at 100%). Values < 100% indicate downregulation and >100% mean upregulation. Data are represented as mean ± SD from three independent experiments. *p*-values are considered statistically significant when * *p* < 0.05, ** *p* < 0.01, and *** *p* < 0.001.

To evaluate the impact of treatments with PTM alone (1 µM, denoted as P1) and the micellar system (F127 (0.125%) + PTM (1 µM), denoted as FP1) on parasite viability, an RT-qPCR analysis was performed. Several key genes were targeted based on their relevance in pathways such as glutathione metabolism, oxidative stress, cell death, membrane transport, and drug resistance. The results revealed a significant, pathway-specific modulation in the expression of several genes depending on the PTM formulation (Figure 8). Regarding glutathione metabolism (Figure 8A–D), both P1 and the micellar system (FP1) exhibited a significant repressive effect on key antioxidant genes compared to the untreated control. P1 significantly downregulated the expression of *γ-GCS* to 65% (*p* = 0.0096) while the FP1 system reduced it to 45% (*p* = 0.0061). A pronounced decrease was also observed for *TR*, with expression levels dropping to 52% (*p* = 0.0225) and 40% (*p =* 0.0005) for P1 and FP1, respectively. Furthermore, both treatments significantly reduced the expression levels of genes encoding peroxidase enzymes relative to the control; *TDPX* gene levels decreased to 68% (*p =* 0.0014) and 55% (*p* = 0.0496), while *TryP* gene expression was reduced to 78% (*p* = 0.0321) and 72% (*p =* 0.0083) for P1 and FP1, respectively. These results suggest that both free and encapsulated PTM may impair the expression levels of genes involved in the parasite’s glutathione-dependent antioxidant defense.

In the oxidative stress response (Figure 8E–G), treatment with P1 resulted in upregulation of *COX4* gene expression to approximately 120% (*p* = 0.0004) compared to the control. A similar change was observed with FP1 compared to the control. Interestingly, FP1 treatment dramatically increased *COX4* gene expression to 148% (*p* = 0.002). In contrast, the expression levels of the *ATPase* gene exhibited a downregulation after exposure to P1 or FP1 compared to the control. Specifically, P1 reduced *ATPase* gene expression levels to 47% (*p* = 0.0162) with respect to those of the control, whereas treatment with FP1 resulted in a more moderate gene downregulation, decreasing its level to 87%. This simultaneous induction of defense-related genes (*COX4*) and repression of genes (*ATPase*) encoding key enzymes suggests that PTM exposure may trigger a complex stress response, involving both compensatory gene activation and the impairment of genes associated with essential energetic or metabolic functions required for parasite homeostasis.

Concerning cell death pathways (Figure 8H,I), both treatments induced a significant transcriptional activation of *MCA5,* a key marker of apoptotic-like death. P1 treatment resulted in an increase in gene expression, reaching approximately 136% (*p* = 0.012) compared to the control. Similarly, the FP1 micellar system also showed a significant increase, raising gene expression to approximately 177% (*p* = 0.0015) compared to the control. Conversely, the *CAPLP* gene, associated with survival mechanisms, was significantly repressed by both medicines. P1 treatment reduced *CAPLP* gene expression levels to approximately 49% (*p* = 0.0016) relative to those of the control, and the FP1 system also significantly decreased those levels to 67% (*p* = 0.0089). These findings suggest that, through PTM treatment (free and encapsulated), a complex cell death activation mechanism may be induced, consistent with the upregulation of genes encoding metacaspases and the suppression of genes associated with survival-related proteases.

Furthermore, the expression of genes involved in membrane transporters was also modulated (Figure 8J,K). P1 treatment resulted in a significant decrease in *SERCA* gene expression to approximately 41% (*p* = 0.0058) compared to the control. The FP1 micellar system also showed a significant decrease in gene expression to approximately 49% (*p* = 0.0185) compared to the control. In contrast, when the expression levels of *AQP1* were assessed, no significant changes were observed with P1 or FP1 treatment.

Finally, the expression of drug resistance genes (*MRPA* and *PRP1*) was analyzed (Figure 8L,M). Both P1 and FP1 systems resulted in a significant decrease in the expression of both genes when compared to the untreated control. Specifically, for the *MRPA* gene, the P1 treatment induced a significant downregulation in gene expression to approximately 58% (*p* = 0.0019) compared to the control. A similar result was observed with *PRP1* expression, which showed a decrease in gene expression with both treatments, 54% (*p* ≤ 0.0001) and 27% (*p =* 0.0002) for P1 and FP1, respectively.

In summary, the gene profiling analysis revealed a complex alteration of gene expression levels, consistent with a beneficial response to PTM, both free and micellar-encapsulated, at the 1 µM concentration (Figure 8). Our findings also suggest that PTM may exert its antileishmanial effect by simultaneously disrupting genes involved in crucial cellular pathways, including the glutathione-dependent antioxidant system and calcium homeostasis, while activating genes related to cell death. Notably, the downregulation of drug-resistance-related genes seems to support the observed drug efficacy too.

## 3. Discussion

The search for innovative formulations for drugs like PTM, which present biopharmaceutical limitations and toxicity, is fundamental for improving their therapeutic efficacy [36]. In this study, the potential of F127 polymeric micelles as nanocarriers for PTM is investigated, utilizing a combination of physicochemical techniques and biological assays. DLS analyses provided information on the size and stability of F127 micelles and how these parameters are influenced by the presence of PTM at different temperatures. At 0.125% concentration, F127 formed stable micelles in PBS, exhibiting a monomodal narrow size profile at 37 °C. Moreover, the micellar size remained largely unaltered by the presence of PTM, confirming the formation of a stable nanostructure at body temperature even at the low polymer concentration used [29,31,37,38]. Furthermore, the emission spectra of fluorescent PTM showed a notable increase in intensity and a blue shift in the presence of micellized F127, suggesting a change in the chemical environment of the drug in the presence of PMs, compatible with a less polar, constrained environment in the micelle, particularly at 37 °C; 1D NMR data confirm this result, as evidenced in the changes in the EO signal of the copolymer in the presence of PTM and in the aromatic moiety of the drug, together with the lack of shifts in the PO ones of F127. This outcome indicates a different magnetic environment of the whole PTM, consistent with its encapsulation in the micelles, fully formed at 37 °C. Overall, the NMR results strongly support that PTM not only partitions into the micellar system but it is lodged predominantly in the hydrophilic domain formed by the PEO blocks (hydrophilic corona) of the F127 micelles, rather than in the hydrophobic core. This trend aligns with the primary advantage of polymeric micelles as drug carriers: their ability to improve the solubility and stability of drugs by encapsulating them within a protective core [30,31,34,38]. These findings are critical since the size of the nanocarrier can directly influence its biological outcomes, including cellular uptake by macrophages and its ability to penetrate tissues [28,36,37]. Initial biological evaluations confirmed the potent but cytotoxic effects of PTM. PTM demonstrated a potent antileishmanial activity against *L. major* promastigotes (IC_50_ = 3.25 ± 0.34 µM). However, the drug also showed cytotoxicity to macrophages (CC_50_ = 2.90 ± 0.77 µM). This narrow selectivity index (SI = CC_50_/IC_50_ < 1) highlights the major clinical limitation of PTM and underscores the critical need for advanced drug delivery systems to improve its safety profile. In contrast, the F127 excipient alone showed almost no toxicity at the concentrations tested, confirming its suitability as a safe nanocarrier for this application [37]. Importantly, the F127 micellar formulation dramatically mitigated the aforementioned cytotoxicity since its CC_50_ value was much higher (9.27 ± 1.31 μM vs. 2.90 ± 0.77 µM). This trend was evidenced by macrophage survival remaining high after treatment with up to 7 µM PTM. This finding strongly supports the capability of F127 micelles to reduce the cytotoxicity of PTM to host cells while effectively preserving its antileishmanial efficacy. The analysis of the dose–response curves highlights a clear therapeutic advantage of encapsulation, creating a therapeutic window that is absent for the free PTM. With an SI value greater than 2, F127-PTM appears to be more selective than PTM alone and may therefore be considered a promising candidate for further development, as it indicates a substantial gap between host cell toxicity and efficacy against *Leishmania* promastigotes.

The gene expression analysis provided crucial molecular insights into the multifaceted mechanism of action of PTM and the effects of the micellar formulation. Our results suggest that both PTM and its micellar formulation may concurrently influence genes related to multiple essential cellular pathways, resulting in the potential disruption of parasite homeostasis. Specifically, the significant downregulation of glutathione metabolism genes, including *γ-GCS* (the rate-limiting enzyme for glutathione synthesis [39,40]), *TR* (essential for maintaining redox balance [12,13,16,41]), and genes encoding antioxidant enzymes *TPDX* [42] and *TryP* [16], may be consistent with the induction of an oxidative stress response in the parasitic cells through the treatment. The FP1 micellar system showed a significant downregulation of the *γ-GCS* gene (*p* < 0.0096) and this should be explored at the protein level to clarify if the F127 formulation may enhance the drug’s effect by increasing its cellular uptake or altering its bioavailability, leading to a more pronounced metabolic disruption. Furthermore, the role of both P1 and FP1 in the induction of a defensive oxidative stress response needs to be investigated and these further results might support the observed upregulation of mitochondrial genes involved in respiratory function like *COX4* [20,43] whereas *FeSOD* [44,45] a gene encoding superoxide dismutase activity remained stable. Both P1 and FP1 treatments caused a significant increase in gene expression levels of *MCA5*—encoding a key effector of apoptosis-like cell death in *Leishmania* [21,46]—suggesting that the role of PTM in actively promoting programmed cell death response is interesting and should be clarified in the future. Additionally, the levels of *CAPLP*, a gene encoding a protease linked to parasite virulence and survival [47,48], was significantly downregulated by both P1 and FP1 treatments, suggesting that proteins that can promote survival and infectivity [22] may be of interest for future studies. This approach can also be extended to membrane transporters, which are vital for maintaining cellular homeostasis [49]. The expression levels of *SERCA*, the gene of an ATPase crucial for calcium regulation [17,18], were significantly downregulated by both treatments (P1 and FP1). It is well known that Ca^2+^ is one of the major signaling molecules in *Leishmania*, controlling processes such as cell invasion, differentiation, and response to stress [49,50]. More experiments will be helpful to elucidate if PTM may disrupt intracellular calcium homeostasis, a process fundamental for parasite metabolism and adaptation. Finally, the expression of drug-resistance-related genes, *MRPA* and *PRP1,* was further analyzed [23,24,51]. The treatments P1 and FP1 resulted in a significant downregulation of these genes relative to the untreated control. While P1 decreased gene expression to 54% (*p* ≤ 0.0001), the treatment with FP1 formulation achieved a more significant alteration, reducing gene expression to ~27%, and this difference (in *PRP1* expression levels, 54% vs. 27%) observed between FP1 and P1 seems relevant. Altogether, these findings suggest that PTM treatment may be consistent with a lower activation of ABC efflux transporters.

This study provides compelling evidence for the potential of F127 micelles as a PTM delivery system. Herein, the abovementioned results demonstrate that the micellar formulation not only mitigates the inherent cytotoxicity of PTM to host cells but also maintains its potent antileishmanial efficacy, resulting in a significantly improved selectivity index. Moreover, gene expression analyses offer deep molecular insights, revealing that the micellar formulation also alters key genes while maintaining or even enhancing the leishmanicidal effects of PTM.

In addition to transcriptional data, those critical changes in gene expression should be validated using complementary approaches, including protein-level analyses (e.g., Western blotting or immunodetection) and functional readouts (e.g., reactive oxygen species generation, drug efflux activity, or apoptosis-related assays). These approaches will be useful for determining whether the drug concurrently disrupts multiple essential parasite pathways and for elucidating the mechanisms by which such disruption occurs. Future research will also focus on evaluating the therapeutic performance of this formulation in *Leishmania* amastigotes, the clinically relevant stage of the parasite, to further validate its potential for cutaneous leishmaniasis treatment.

## 4. Materials and Methods

### 4.1. Chemicals and Reagents

Pluronic^®^ F127 (F127, Sigma-Aldrich, St. Louis, MO, USA), phosphate-buffered saline (PBS) (Sigma-Aldrich, St. Louis, MO, USA), and pentamidine isethionate (PTM, Glentham Life Sciences, Wiltshire, UK), were obtained from their respective suppliers. All chemicals were of analytical grade and used without further purification.

### 4.2. Physicochemical Characterization

#### 4.2.1. Dynamic Light Scattering (DLS)

Micelle size and size distribution were measured with a DynaPro-MS/X photon correlation spectrometer (Wyatt Technology LLC, Santa Barbara, CA, USA) equipped with an 822 nm laser operating at a fixed scattering angle of 90°. Temperature was controlled using an integrated Peltier unit (0.1 °C accuracy). Intensity-weighted distributions were calculated from the autocorrelation functions using DynaLS version 2.8.2 (Alango Ltd., Tirat Carmel, Israel), accounting for the viscosity and refractive index of the corresponding solvent (water or PBS). Samples were filtered through 0.10 µm and 0.22 µm PVDF syringe filters prior to the measurements.

#### 4.2.2. UV–Visible Spectroscopy

UV–visible absorption spectra were acquired with an Agilent Cary 8454 Spectrophotometer (Agilent, East Lyme, CT, USA) using 1 cm quartz cuvettes. PTM solutions (0.01 mM) prepared in PBS were analyzed in the absence and presence of F127 at 37 °C. The spectra were recorded from 200 nm to 380 nm, at 1 nm intervals.

#### 4.2.3. Fluorescence Spectroscopy

Fluorescence emission spectra were recorded using an Edinburgh FLS920 spectrofluorometer (Edinburgh Instruments Ltd., Livingston, UK) equipped with a thermostated cell holder. PTM solutions were prepared in PBS alone or supplemented with 0.5% or 1% F127. Measurements were conducted at 21 °C and 37 °C. Excitation wavelength was set at 265 nm and emission spectra were collected from 280–420 nm with 5 nm slit widths for both excitation and emission.

#### 4.2.4. Nuclear Magnetic Resonance Spectroscopy (NMR)

^1^H NMR and 2D-NOESY spectra were recorded on a Bruker Avance Neo 400 MHz spectrometer (Bruker Corporation, Billerica, MA, USA). Samples were prepared in D_2_O (>99.9% deuteration, Sigma-Aldrich) and the temperature adjusted to ensure micelle formation of F127 (37 °C). Standard Bruker pulse sequences were used, and data were processed with TopSpin 4.0.

### 4.3. Biological Evaluation

#### 4.3.1. Cells and Culture Conditions

*Leishmania major* promastigotes (strain Lv39c5, Rho/SU/59/P) were cultured at 26 °C in M199 medium supplemented with 25 mM HEPES (pH 7.2), 0.1 mM adenine, 0.0005% (*w*/*v*) hemin, 2 mg/mL biopterin, 0.0001% (*w*/*v*) biotin, 10% (*v*/*v*) heat-inactivated fetal bovine serum (FBS), and an antibiotic cocktail (50 U/mL penicillin, 50 mg/mL streptomycin). To preserve virulence, parasites were isolated from infected BALB/c mouse spleen and maintained for no more than five in vitro passages.

RAW264.7 (ATCC-TIB 71) macrophages were cultured at 37 °C and 5% CO_2_ in DMEM supplemented with 10% FBS and penicillin–streptomycin, as previously described [30]. Adherent cells were detached by gentle scraping and used immediately for cytotoxicity assays.

#### 4.3.2. Cytotoxicity Assay on Macrophages

Cytotoxicity was assessed using the MTT colorimetric assay. RAW264.7 cells (2 × 10^4^ cells/well) were seeded in 96-well plates and allowed to adhere for 5 h at 37 °C in a 5% CO_2_ humidified atmosphere. Cells were then exposed to increasing concentrations of PTM (0.94–15 μM) or F127 (0.008–2% *w*/*v*). After 48 h of incubation, 20 μL of MTT solution (5 mg/mL in PBS) was added to each well and incubated for 4 h under the same conditions. Formazan crystals were solubilized with 100 μL DMSO, and the absorbance was measured at 540 nm using a Multiskan EX microplate reader (Thermo Fisher Scientific Inc., Waltham, MA, USA) Cell viability was calculated relative to untreated cells. The “50% cytotoxic concentration” (CC_50_) indicates the concentration of a drug that causes a 50% reduction in cell viability or survival in host cells (macrophages). CC_50_ values were determined using non-linear regression in GraphPad Prism 10.4.1. All assays were performed at least in triplicate, and data represent the mean (±SD).

#### 4.3.3. Activity Against Promastigotes

*L. major* promastigotes in the exponential growth phase were adjusted to 2.5 × 10^6^ cells/mL in M199 medium. Subsequently, 100 µL of the stock solution was seeded in 96-well plates with increasing concentrations of the compounds, as described above, and maintained at 26 °C. After 48 h of incubation, 20 µL of MTT was added per well and the plates were incubated for 4 h under the same conditions. Then, 80 µL of DMSO was added to each well to dissolve formazan crystals. The “50% inhibitory concentration” (IC_50_) is the concentration of drug that reduces cell (leishmania) survival or viability by 50%. The optical density and the IC_50_ value were also measured and calculated as described above. These assays were conducted at least in triplicate in independent experimental runs.

To evaluate the safety profile of the treatments, the selectivity index (SI) was calculated. The SI represents the ratio of the cytotoxicity to host cells to the antileishmanial activity, determined using the following formula: SI = CC_50_/IC_50_. Values below 1 suggest the drug harms host cells while a value of 1 means equal toxicity and efficacy. Higher SI values (>1) are better, indicating a gap between toxicity to host cells and efficacy against leishmania parasites.

#### 4.3.4. RNA Extraction and cDNA Synthesis

*L. major* promastigotes were incubated for 24 h with increasing compound concentrations at 26 °C. Total RNA was extracted using the Maxwell^®^ RSC simplyRNA Tissue Kit (Promega, Madison, WI, USA). RNA purity and concentration were assessed by a NanoDrop One spectrophotometer (A260/A280 = 2.0–2.1; A260/A230 = 2.1–2.4). cDNA was synthesized from 1 µg RNA using the Reverse Transcriptase M-MLV Invitrogen™ (Thermo Fisher Scientific Inc., Waltham, MA, USA), primed with random hexamers, under the following conditions: 37 °C for 60 min, 90 °C for 1 min, and 4 °C hold. cDNA was stored at −20 °C.

#### 4.3.5. Gene Expression Profiling by RT-qPCR

Genes involved in redox metabolism (*γ-GCS*, *TR*, *TDPX*, *TryP*), membrane transport (*SERCA*, *AQP1*), oxidative stress (*ATPase*, *COX4*, *FeSOD*), cell death pathways (*MCA5*, *CAPLP*), and drug resistance (*MRPA*, *PRP1*) were selected for expression analysis. Primers for these targets and the reference gene *gapdh* were designed using Benchling and validated with NCBI Primer-BLAST (https://www.ncbi.nlm.nih.gov/tools/primer-blast/ (accessed on 24 January 2026)). Primer characteristics included: Tm 58–62 °C, GC content 40–60%, amplicon size 80–200 bp. A melt curve confirmed specificity. The gene-specific primers used for amplification are shown in Table 1.

RT-qPCRs were performed using iQ SYBR^®^ Green Supermix (Cat. No. 1708882) on a CFX Connect™ Real-Time PCR Detection System (both from Bio-Rad Laboratories, Inc., Hercules, CA, USA) in 10 µL volumes containing 1 µL of cDNA, 10 µM primers, and nuclease-free water. Cycling parameters were 95 °C for 3 min, 35 cycles of 95 °C for 15 s, 60 °C for 15 s, and 72 °C for 25 s; followed by a melt curve from 72–95 °C (0.5 °C/s). Samples were run in triplicate, with all reactions performed in triplicate, including no-template controls.

Gene expression was expressed as fold-change percentage (FC%), normalized to untreated controls (100%). FC% = 100 × final value/control value. Accordingly, FC% > 100% indicates upregulation and <100% downregulation.

### 4.4. Statistical Analyses

Data are presented as mean ± SD. Statistical significance of gene expression changes was assessed using unpaired two-tailed *t*-tests. Significance thresholds were set at *p* < 0.05 (*), *p* < 0.01 (**), and *p* < 0.001 (***). Analyses were performed using GraphPad Prism version 10.4.1 and OriginPro 2025.

## 5. Conclusions

This research demonstrates the potential of F127 polymeric micelles as a highly viable nanocarrier for PTM, effectively addressing the significant toxicity and biopharmaceutical limitations of the free drug. Physicochemical analyses, including DLS, UV-vis, fluorescence emission spectra, and NMR, confirmed the formation of a stable micellar system and the successful encapsulation of PTM in the PEO shell. This stable nanostructure further exhibits thermoresponsive behavior, forming a thermogel at a high copolymer concentration and body temperature, which is advantageous for the topical delivery of the drug as a thermogel. Interestingly, the biological evaluations demonstrated that the micellar formulation not only maintained PTM’s potent antileishmanial activity but also exhibited a significantly improved safety profile. Gene expression analyses shed some light on the potential molecular rationale for this enhanced efficacy, revealing a downregulation of drug resistance genes along with the alteration of the expression levels of several genes encoding molecules involved in critical pathways in the parasite such glutathion metabolism, mitochondrial function, and cell death. Additional experiments, including biological evaluation in amastigotes as well as protein-level and functional validations, should be conducted in future studies to establish clinical relevance and to achieve a deeper understanding of the underlying biological mechanisms. Collectively, these results propose an interesting base for the potential use of this stable nanoformulation (PTM-loaded F127 micellar system) in the future as a promising therapeutic strategy for the treatment of leishmaniasis and an approach to continue improving drug efficacy and safety.

## Figures and Tables

**Table 1:** Sequence of primers used for real-time PCR.

Gene	Primer Sequence: Forward	Primer Sequence: Reverse
*Aquaglyceroporin 1* (*AQP 1*)	TTCCTATGGCCTCAACCCCGCA	CGAAAAGGGCGCCAACAAACGG
*ATPase putative* (*ATPase*)	TGAGCTCTGCGGCGGTGAAAAG	CGTACGGAAACTGGGGCACACC
*Calcium-translocating P-type ATPase* (*SERCA*)	CCGATTCAGCTCTTGTGGGT	CAACAATGGGCTCGTCTCCT
*Calpain protease-like protein* (*CAPLP*)	CGCCGACCTGTACAAGCTAA	ACTGTGAAGAGCAGAGCACC
*Cytochrome c oxidase subunit IV* (*COX4*)	GTTGGCTGAGGACAACCGCCTC	CCGTGTTAAGGTTCCAGCGCGT
*Gamma-glutamylcysteine synthetase* (*γ-GCS*)	TCAGCCAACGCCGTTCGAGAAC	CGATGTGCGCGGCCCATATTCT
*Glutathione peroxidase* (*TDPX*)	TCACCGTTCTCGCGTTTCCGTG	GGGTCGGCTGAGGAACCCTTCA
*Iron superoxide dismutase (Fe-SOD*)	AGGGCATGTCGAAGGAGCAGGT	TTCGACGCAAGAGCCGAGTTCG
*Multidrug-resistant protein A* (*MRPA*)	ATGGCGACACCAGACTTTGT	CTGCGAGGGAGCATGGTTTA
*Pentamidine Resistance Protein 1* (*PRP1*)	GAACGAGCTGTCACGTGTGGGG	TACAACGGCAGCGCACTGTCAG
*Putative metacaspase* (*MCA5*)	GCAACGGGTTACCCAGTCCACG	GACACGCCAAGAGTTGCCTGCT
*Trypanothione reductase* (*TR*)	ACGAAGAACGAGGACGGCTCGA	CTTTGCTGTTTGAACGCCGGCC
*Tryparedoxin peroxidase* (*TRYPall*)	CAGCGTGGAGGAGGTTCTAC	CTCGACAGACGCATTCGGTT

## Data Availability

The original contributions presented in this study are included in the article. Further inquiries can be directed to the corresponding author(s).

## References

[1] Aronson N., Herwaldt B.L., Libman M., Pearson R., Lopez-Velez R., Weina P., Carvalho E.M., Ephros M., Jeronimo S., Magill A. (2016). Diagnosis and Treatment of Leishmaniasis: Clinical Practice Guidelines by the Infectious Diseases Society of America (IDSA) and the American Society of Tropical Medicine and Hygiene (ASTMH). Clin. Infect. Dis..

[2] Burguete-Mikeo A., Fernández-Rubio C., Peña-Guerrero J., El-Dirany R., Gainza L., Carasa Buj B., Nguewa P.A. (2023). Characterization of *Leishmania* parasites isolated from naturally infected mammals. Animals.

[3] World Health Organization Leishmaniasis. https://www.who.int/news-room/fact-sheets/detail/leishmaniasis.

[4] Hailu A., Dagne D.A., Boelaert M., Gyapong J., Boatin B. (2016). Leishmaniasis. Neglected Tropical Diseases–Sub-Saharan Africa.

[5] Carvalho S.H., Frézard F., Pereira N.P., Moura A.S., Ramos L.M.Q.C., Carvalho G.B., Rocha M.O. (2019). American tegumentary leishmaniasis in Brazil: A critical review of the current therapeutic approach with systemic meglumine antimoniate and short-term possibilities for an alternative treatment. Trop. Med. Int. Health.

[6] Salloum T., Tokajian S., Hirt R.P. (2021). Advances in Understanding Leishmania Pathobiology: What Does RNA-Seq Tell Us?. Front. Cell Dev. Biol..

[7] Dubie T., Mohammed Y. (2020). Review on the Role of Host Immune Response in Protection and Immunopathogenesis during Cutaneous Leishmaniasis Infection. J. Immunol. Res..

[8] Paton H., Sarkar P., Gurung P. (2025). An overview of host immune responses against Leishmania spp. infections. Hum. Mol. Genet..

[9] Liese J., Schleicher U., Bogdan C. (2008). The innate immune response against Leishmania parasites. Immunobiology.

[10] Maspi N., Abdoli A., Ghaffarifar F. (2016). Pro- and anti-inflammatory cytokines in cutaneous leishmaniasis: A review. Pathog. Glob. Health.

[11] Panahi E., Stanisic D., Peacock C., Herrero L. (2021). Protective and Pathogenic Immune Responses to Cutaneous Leishmaniasis.

[12] Battista T., Colotti G., Ilari A., Fiorillo A. (2020). Targeting Trypanothione Reductase, a Key Enzyme in the Redox Trypanosomatid Metabolism, to Develop New Drugs against Leishmaniasis and Trypanosomiases. Molecules.

[13] Turcano L., Torrente E., Missineo A., Andreini M., Gramiccia M., Muccio T.D., Genovese I., Fiorillo A., Harper S., Bresciani A. (2018). Identification and binding mode of a novel Leishmania Trypanothione reductase inhibitor from high throughput screening. PLoS Negl. Trop. Dis..

[14] Madia V.N., Ialongo D., Patacchini E., Exertier C., Antonelli L., Colotti G., Messore A., Tudino V., Saccoliti F., Scipione L. (2023). Inhibition of Leishmania infantum Trypanothione Reductase by New Aminopropanone Derivatives Interacting with the NADPH Binding Site. Molecules.

[15] Amiri-Dashatan N., Koushki M., Rezaei-tavirani M., Ahmadi N. (2020). Stage-Specific Differential Gene Expression of Glutathione Peroxidase in Leishmania Major and Leishmania Tropica. Rep. Biochem. Mol. Biol..

[16] Das S., Saha T., Yadav S., Shaha C. (2022). A Novel Role of Secretory Cytosolic Tryparedoxin Peroxidase in Delaying Apoptosis of Leishmania-Infected Macrophages. Mol. Cell. Biol..

[17] Meade J.C. (2019). P-type transport ATPases in Leishmania and Trypanosoma. Parasite.

[18] Paul R., Banerjee S., Sen S., Dubey P., Maji S., Bachhawat A.K., Datta R., Gupta A. (2022). A novel leishmanial copper P-type ATPase plays a vital role in parasite infection and intracellular survival. J. Biol. Chem..

[19] Figarella K., Uzcategui N.L., Zhou Y., LeFurgey A., Ouellette M., Bhattacharjee H., Mukhopadhyay R. (2007). Biochemical characterization of Leishmania major aquaglyceroporin LmAQP1: Possible role in volume regulation and osmotaxis. Mol. Microbiol..

[20] Li Y., Park J.S., Deng J.H., Bai Y. (2006). Cytochrome c oxidase subunit IV is essential for assembly and respiratory function of the enzyme complex. J. Bioenerg. Biomembr..

[21] Ambit A., Fasel N., Coombs G.H., Mottram J.C. (2008). An essential role for the Leishmania major metacaspase in cell cycle progression. Cell Death Differ..

[22] Ennes-Vidal V., Vitório B.d.S., Menna-Barreto R.F.S., Pitaluga A.N., Gonçalves-da-Silva S.A., Branquinha M.H., Santos A.L.S., d’Avila-Levy C.M. (2019). Calpains of Leishmania braziliensis: Genome analysis, differential expression, and functional analysis. Mem. Inst. Oswaldo Cruz.

[23] Coelho A.C., Beverley S.M., Cotrim P.C. (2003). Functional genetic identification of PRP1, an ABC transporter superfamily member conferring pentamidine resistance in Leishmania major. Mol. Biochem. Parasitol..

[24] Moncada-Diaz M.J., Rodríguez-Almonacid C.C., Quiceno-Giraldo E., Khuong F.T.H., Muskus C., Karamysheva Z.N. (2024). Molecular Mechanisms of Drug Resistance in *Leishmania* spp.. Pathogens.

[25] Nguewa P.A., Fuertes M.A., Cepeda V., Iborra S., Carrión J., Valladares B., Alonso C., Pérez J.M. (2005). Pentamidine is an antiparasitic and apoptotic drug that selectively modifies ubiquitin. Chem. Biodivers..

[26] Hafiz S., Kyriakopoulos C. (2025). Pentamidine. StatPearls.

[27] Gu T., Tian X., Wang Y., Yang W., Li W., Song M., Zhao R., Wang M., Gao Q., Li T. (2023). Repurposing pentamidine for cancer immunotherapy by targeting the PD1/PD-L1 immune checkpoint. Front. Immunol..

[28] Registre C., Soares R.D.O.A., Rubio K.T.S., Santos O.D.H., Carneiro S.P. (2023). A Systematic Review of Drug-Carrying Nanosystems Used in the Treatment of Leishmaniasis. ACS Infect. Dis..

[29] Cagel M., Tesan F.C., Bernabeu E., Salgueiro M.J., Zubillaga M.B., Moretton M.A., Chiappetta D.A. (2017). Polymeric mixed micelles as nanomedicines: Achievements and perspectives. Eur. J. Pharm. Biopharm..

[30] Dirany Z., Smith G.N., Aydillo C., Nguewa P., González-Gaitano G. (2024). Structure and activity of amphiphilic PEO-PPO-based polymeric micelles and gels incorporating host–guest complexes of miltefosine as novel formulations for the treatment of leishmaniasis. J. Mol. Liq..

[31] Tavares G.S.V., Mendonça D.V.C., Miyazaki C.K., Lage D.P., Soyer T.G., Carvalho L.M., Ludolf F. (2019). A Pluronic^®^ F127-based polymeric micelle system containing an antileishmanial molecule is immunotherapeutic and effective in the treatment against Leishmania amazonensis infection. Parasitol. Int..

[32] Mendonça D.V.C., Tavares G.S.V., Lage D.P., Soyer T.G., Carvalho L.M., Dias D.S., Ribeiro P.A., Ottoni F.M., Antinarelli L.M., Vale D.L. (2019). In vivo antileishmanial efficacy of a naphthoquinone derivate incorporated into a Pluronic^®^ F127-based polymeric micelle system against Leishmania amazonensis infection. Biomed. Pharmacother..

[33] Wei Z., Hao J., Yuan S., Li Y., Juan W., Sha X., Fang X. (2009). Paclitaxel-loaded Pluronic P123/F127 mixed polymeric micelles: Formulation, optimization and in vitro characterization. Int. J. Pharm..

[34] Chavoshy F., Makhmalzade B. (2018). Polymeric micelles as cutaneous drug delivery system in normal skin and dermatological disorders. J. Adv. Pharm. Technol. Res..

[35] Lupu A., Gradinaru L.M., Rusu D., Bercea M. (2023). Self-Healing of Pluronic^®^ F127 Hydrogels in the Presence of Various Polysaccharides. Gels.

[36] Andreana I., Bincoletto V., Milla P., Dosio F., Stella B., Arpicco S. (2022). Nanotechnological approaches for pentamidine delivery. Drug Deliv. Transl. Res..

[37] Naharros-Molinero A., Caballo-González M.Á., De La Mata F.J., García-Gallego S. (2022). Direct and Reverse Pluronic Micelles: Design and Characterization of Promising Drug Delivery Nanosystems. Pharmaceutics.

[38] Ghezzi M., Pescina S., Padula C., Santi P., Del Favero E., Cantù L., Nicoli S. (2021). Polymeric micelles in drug delivery: An insight of the techniques for their characterization and assessment in biorelevant conditions. J. Control. Release.

[39] Mukherjee A., Roy G., Guimond C., Ouellette M. (2009). The γ-glutamylcysteine synthetase gene of Leishmania is essential and involved in response to oxidants. Mol. Microbiol..

[40] Hejazi S.H., Saberi S., Arjmand R., Soleimanifard S. (2021). The study of P-glycoprotein A, G-glutamylcysteine synthetase 1, and aquaglyceroporin 1 genes expression in non-healing zoonotic cutaneous leishmaniasis cases. J. Shahrekord Univ. Med. Sci..

[41] Sarfraz M., Bakht M.A., Alshammari M.S., Alrofaidi M., Alzahrani A.R., Eltaib L., Asdaq S.M., Aba Alkhayl F.F., Abida Imran M. (2025). Beyond traditional medications: Exploring novel and potential inhibitors of trypanothione reductase (LmTr) of Leishmania parasites. J Biomol Struct Dyn..

[42] Handy D.E., Lubos E., Yang Y., Galbraith J.D., Kelly N., Zhang Y.Y., Leopold J.A., Loscalzo J. (2009). Glutathione Peroxidase-1 Regulates Mitochondrial Function to Modulate Redox-dependent Cellular Responses. J. Biol. Chem..

[43] Shaheen F., Stephany-Brassesco I., Kelly B.L. (2021). Dynamic modulation of Leishmania cytochrome c oxidase subunit IV (LmCOX4) expression in response to mammalian temperature. Mol. Biochem. Parasitol..

[44] Mittra B., Laranjeira-Silva M.F., Miguel D.C., Perrone Bezerra de Menezes J., Andrews N.W. (2017). The iron-dependent mitochondrial superoxide dismutase SODA promotes Leishmania virulence. J. Biol. Chem..

[45] Araújo J.S.C., Oliveira L.d.M., Andrade K.V.F.d., Benevides R.G., Leite F.H.A., Junior M.C.d.S. (2023). Superoxide Dismutase Inhibitors against Malaria, Leishmaniasis, and Chagas Disease: Systematic Review. Curr. Drug Targets.

[46] Basmaciyan L., Casanova M. (2019). Cell death in Leishmania. Parasite.

[47] Marinho F.A., Gonçalves K.C.S., Oliveira S.S.C., Gonçalves D.S., Matteoli F.P., Seabra S.H., Oliveira A.C., Bellio M., Oliveira S.S., Souto-Padron T. (2014). The Calpain Inhibitor MDL28170 Induces the Expression of Apoptotic Markers in Leishmania amazonensis Promastigotes. PLoS ONE.

[48] Ennes-Vidal V., Menna-Barreto R.F.S., Branquinha M.H., Santos A.L.S.D., D’avila-Levy C.M. (2017). Why calpain inhibitors are interesting leading compounds to search for new therapeutic options to treat leishmaniasis?. Parasitology.

[49] Akpunarlieva S., Burchmore R. (2017). The role of membrane transporters in Leishmania virulence. Emerg. Top. Life Sci..

[50] Moreno S.N., Docampo R. (2003). Calcium regulation in protozoan parasites. Curr. Opin. Microbiol..

[51] Légaré D., Cayer S., Singh A.K., Richard D., Papadopoulou B., Ouellette M. (2001). ABC Proteins of Leishmania. J. Bioenerg. Biomembr..

